# The Science and the Art of Feeding

**Published:** 1903-10-10

**Authors:** 


### The Hospital.

A Journal of

The Medical Sciences and Hospital Administration.

Vol. XXXV.—No. 889. OCTOBER 10, 1903.

### The Science and the Art of Feeding.

The question of food and feeding is one which at the present day is very much in evidence. The crank and the faddist are abroad in the land, and the virtues of new foods, or rather of new names, are proclaimed in decorative protests from every hoarding. Even within the ranks of medicine are to be found some who announce that health on the one hand and disease on the other depend solely on the practice or the neglect of certain rigid dietetic restrictions. Standing on a somewhat different platform is the claim of the physiologist to select and prescribe the food habits of the race. By chemical calculations, by laboratory experiments, and by other similar methods, physiology has determined man's necessities in relation to carbon, to nitrogen, and to other elements, and has endeavoured to suggest the most desirable and economical sources from which these needs may be supplied. The proceeding is a very plausible one, and when presented with appropriate diagrams invariably commands the unstinted applause of a popular audience. Yet it is notorious that while the advice of the physiologist runs counter to many dietetic customs, these customs continue to flourish. The demonstration so convincing in theory is steadily disregarded in practice. Men continue to eat and to drink according to their own good pleasure, and in spite alike of the advice and the warnings of physiological science. In these circumstances it seems reasonable to inquire whether the claims of physiology in this respect are well founded. A superficial glance at the question will probably provoke an affirmative response. Surely, it will be said, of all guides to dietetic wisdom none is likely to be so safe as he who speaks with expert knowledge of the wants and functions of the body. The theory of nutrition is an important and essential part of his studies, and to his rules therefore adherence may confidently be given. But a little more reflection may affordreasons for modifying this assent. For it is obvious that during countless thousands of years man pursued the successful practice of dietetics without the slightest tincture of physiological knowledge. It is hardly possible in view of the doctrine of the survival of the fittest to suppose that habits and practices thus evolved are other than beneficial both to the individual and to the race. If this is so it follows that food habits deliberately practised by large bodies of men can claim the impressive sanction of a prolonged and successful experience. This position is further strengthened by known facts regarding the food habits of the lower animals. No one questions that these, under the influence of the cosmic struggle, have been guided to wise and beneficial issues. On what ground, then, can it be supposed that man appears as the solitary exception to a universal law and stands condemned as the one animal who has gone astray from the path of dietetic prudence? If physiology and experience are to be quoted against each other it is not a foregone conclusion that the voice of the latter must subside into silence. Again, it must be remembered that not all the purposes of food are susceptible of estimate by the tests of the physiologist. He may, it is true, group and classify foods in reference to their tissue-forming or energy-yielding functions, but there are subtle ends to be served for which, at present at least, he has no standards. Thus it can hardly be doubted that in the development of certain mental qualities which have most directly subserved the progress of the race food habits have played an important part. And there need be little hesitation in suggesting that were, for example, the Anglo-Saxon peoples promptly to accommodate their habits to the claims of what physiology would define as dietetic righteousness, the racial characteristics of succeeding generations would be profoundly modified. It is equally obvious, unless the beneficial trend of evolution is to be denied, that such modifications would not be in the direction of improvement. This consideration introduces another element where scientific tests fail. Experimental work in the laboratory is concerned with individuals. It may show what foods promote growth, and weight, and muscular capacity, and may suggest by inference that such foods favour health and long life. But this is too narrow a view from which to attempt the solution of the food problem. The weight and height of the individual, even his good health and long life, do not mean necessarily the welfare of the race. It may be essential that the individual should "wither" in order that "the world" may be "more and more." Good health and long life are not ends in themselves. They are valuable only because they mean opportunities for work and achievement. It is not long life but strenuous life that makes "the great world spin for ever down the ringing grooves of change." Those food habits are valuable, therefore, which promote national energy, and enterprise, and force of character, qualities which do not respond to scientific measurement. It is not here contended that a science of dietetics is of no value. On the contrary, the study of recognised food habits may provide both interesting and useful information. It may determine what share each habit plays in the promotion of human well-being. In some it may discover that evil is mixed with good, and may be able to suggest means by which the benefit may be obtained without alloy. But the method must be inductive, not deductive. It must start from the position that in what is practised by large numbers of men free to choose, there is without doubt some element of value. The accumulated experiences of innumerable generations are not to be dismissed by the formulas of the laboratory. It is not these latter alone which can claim an experimental basis. What man has learned in the struggle for existence has a position not less secure than the conclusions of the physiological chemist. The latter may define food values, or some of them, in terms of science, but he cannot successfully set his analyses and experiments against customs which are the outcome of the matured experiences of the race. It is with these that the final word rests. There remains for the physiologist a task of much interest. His function, in brief, is not to prescribe our habits but to explain them.

